# An Associative Analysis of Recognition Memory: Relative Recency Effects in an Eye-Tracking Paradigm

**DOI:** 10.1037/xan0000258

**Published:** 2020-07

**Authors:** Aleksander W. Nitka, Charlotte Bonardi, Jasper Robinson

**Affiliations:** 1 School of Psychology, University of Nottingham

**Keywords:** recognition, eye-tracking, recency, object-in-place, association

## Abstract

We report 2 eye-tracking experiments with human variants of 2 rodent recognition memory tasks, *relative recency* and *object-in-place*. In Experiment 1 participants were sequentially exposed to 2 images, *A* then *B*, presented on a computer display. When subsequently tested with both images, participants biased looking toward the first-presented image *A*: the relative recency effect. When contextual stimuli *x* and *y*, respectively, accompanied *A* and *B* in the exposure phase (*xA*, *yB*), the recency effect was greater when *y* was present at test, than when *x* was present. In Experiment 2 participants viewed 2 identical presentations of a 4-image array, *ABCD*, followed by a test with the same array, but in which one of the pairs of stimuli exchanged position (***BA***CD or *AB***DC**). Participants looked preferentially at the displaced stimulus pair: the object-in-place effect. Three further conditions replicated Experiment 1’s findings: 2 pairs of images were presented one after the other (*AB* followed by *CD*); on a test with *AB* and *CD*, relative recency was again evident as preferential looking at *AB*. Moreover, this effect was greater when the positions of the first-presented *A* and *B* were exchanged between exposure and test (***BA***CD), compared with when the positions of second-presented *C* and *D* were exchanged (*AB***DC**). The results were interpreted within the theoretical framework of the Sometime Opponent Process model of associative learning (Wagner, 1981).

Recognition memory is a key aspect of human declarative memory, and may be defined as the ability to discriminate between items that have been previously encountered from those that have not. The fact that recognition not only declines with age, but can also be selectively impaired in some dementias, has added to an already substantial interest in the phenomenon. In animals, recognition is typically studied in the spontaneous object recognition procedure (Ennaceur & Delacour, 1988), a paradigm that relies on rodents’ natural curiosity, and proclivity to explore their environment and novel items that appear within it. In a typical version of this task, animals are allowed to explore a pair of identical junk objects in an experimental arena for a short period. After a retention interval they are returned to the apparatus, and presented with two further objects, one which they have seen before, and another, novel test item. Greater exploration of the novel object is taken as evidence that the previously encountered one has been recognized. Yet, despite the simplicity of the effect itself, its theoretical basis remains in debate. Some argue that recognition comprises two independent underlying processes, recollection and familiarity (e.g., Aggleton & Brown, 2006; cf. Yonelinas, 2002), while others conceptualize varied performance on object recognition tasks as being perceptual, rather than mnemonic (Cowell, Bussey, & Saksida, 2006). There is also a prevailing tendency to propose explanations of recognition memory that rest heavily on the underlying neural mechanism, and the effect of damage to structures such as hippocampus and perirhinal cortex on recognition memory performance. Moreover, despite the fact that one could view recognition memory as requiring some kind of learning—particularly when it is sustained over long retention intervals—none of these accounts make any direct link with extant learning models.

In view of these issues, several authors (Honey & Good, 2000; Robinson & Bonardi, 2015; Sanderson & Bannerman, 2011) have proposed an alternative account of recognition memory, based on the account of associative learning formulated by Wagner—Sometimes Opponent Process (SOP; Wagner, 1981; Wagner & Brandon, 1989). This is a theory of association formation that also incorporates a conceptualization of stimulus representation. According to this account, every stimulus may be represented as a set of stimulus elements corresponding to its various features, and these elements may occupy one of three different activation states. When a stimulus is presented for the first time, a probabilistically selected proportion of its elements enter a state of primary activation called A1—equivalent to being in the focus of attention, where they can elicit a strong response. However, A1 is of a limited capacity, and so these stimulus elements will decay rapidly into a secondary, larger-capacity activation state termed A2, before returning more slowly to their resting inactive state, I. Compared with A1, A2 is more akin to the periphery of attention, and elements in A2 typically elicit a much weaker response. A key feature of this model is that stimulus elements must complete this cycle before they are able to enter A1 again. Thus, if a stimulus is presented twice in quick succession, on the second presentation many of its elements persist in the A2 state and will be unavailable for reactivation into A1—meaning the response to the stimulus will be weaker on the second presentation than the first. This process, whereby presentation of a stimulus can render subsequent response to that same stimulus less effective, is termed *self-generated priming*.

The model accounts for associative learning by asserting that if two stimuli are presented such that both of them have elements in the A1 state, an excitatory association forms between them; as a result, when one of the stimuli is subsequently presented it can activate elements of its associate directly into the A2 state—a process called *retrieval-generated priming*. Conversely, if the conditioned stimulus (CS) is in the A1 state and the unconditioned stimulus (US) is in A2, an inhibitory association forms between the CS and the US, and as a result the CS prevents US elements from entering A2.

If we assume that the response to the stimuli in spontaneous object recognition is exploration, then this analysis suggests at least two potential mechanisms for performance on this task. Provided the retention interval between the initial, sample presentation and the test is sufficiently short, some of the sample object’s elements will still be in A2 at the point of test, thus reducing responding to this familiar stimulus via the self-generated priming mechanism. In addition, during the sample stage associations may form between elements of the object and the surrounding context; thus, on test the context can prime elements of the preexposed object into their A2 states, and reduce responding to this familiar object even further via retrieval-generated priming. Both mechanisms will reduce responding to the preexposed object relative to that of the novel item, whose elements can freely enter their A1 states.

These explanations of recognition in terms of self-generated and retrieval-generated priming can also account for performance on two common rodent variants of the spontaneous object recognition task, *relative recency* (e.g., Mitchell & Laiacona, 1998) and *object-in-place* (e.g., Dix & Aggleton, 1999). In the relative recency task, preexposure to object *A* is followed after a delay by preexposure to object *B*. At test animals are given a choice between objects *A* and *B*. Selective exploration of first-presented object *A* is typically observed, suggesting sensitivity to the fact that it is less recent. This performance is easy to explain in terms of *self-generated priming*: at test elements of object *A* will have had more time to complete their cycle of decay, and return to the inactive state, than those of object *B*; thus, more of *A*’s elements will be available for recruitment into their A1 states at test, and will elicit a strong exploration response. In one variant of the object-in-place task, animals are exposed to an array of four objects, *ABCD*; at test they are presented with the same four objects, but two of them, *AB*, have exchanged position, while the remaining two, *CD*, have not; the typical observation is that animals are more likely to explore the displaced objects, revealing sensitivity to the place in which the item was originally presented. This result is also easy to explain in terms of SOP, but this time by appealing to the second, *retrieval-generated* priming mechanism. During the sample phase, associations will form between each of the objects and the contextual cues that surround it (e.g., the features of the arena in which rodent is placed). Thus, at test, when the animal is in the part of the arena in which *C* and *D* appeared during the sample phase, the context will prime elements of those objects directly into their A2 states, eliciting only a weak exploration response. In contrast, when the animal approaches the displaced objects, *A* and *B*, the surrounding contextual cues—being different from those that accompanied these objects in preexposure—will be less able to prime A and B’s elements, leaving them better able to enter their A1 states from their I states and provoking a strong exploration response.

The spontaneous object recognition task and these variants are widely used in animal work. In contrast, although there is a vast literature on recognition memory in human participants, much of this work uses explicit judgments as an index of recognition memory (yes/no or forced-choice recognition tests), and relatively little behavioral measures comparable with those used in animal studies. The exception is in the rather rare assessment of recognition memory using eye-tracking. Some studies have used eye-tracking in human versions of the spontaneous object recognition (termed the visual paired comparison task, or VPC; Crutcher et al., 2009; Gills, Glenn, Madero, Bott, & Gray, 2019; Whitehead et al., 2018; Zola, Manzanares, Clopton, Lah, & Levey, 2013), and object-in-place tasks (Hannula, Ryan, Tranel, & Cohen, 2007; Mahoney, Kapur, Osmon, & Hannula, 2018; Richmond & Nelson, 2009; Ryan & Cohen, 2004; Yeung et al., 2019), although the latter have yielded somewhat mixed results. However, we are unaware of any demonstrations of relative recency using eye-tracking, and so to provide such a demonstration was the first aim of the present experiments. The second was to evaluate our SOP interpretation of these phenomena, which has been the subject of object recognition experiments with rodents (e.g., Tam, Bonardi, & Robinson, 2015; Tam, Robinson, Jennings, & Bonardi, 2014; Whitt, Haselgrove, & Robinson, 2012; Whitt & Robinson, 2013).

## Experiment 1

In Experiment 1 participants received three types of relative recency task, in which they were first exposed to a sample presentation of target image *A*, followed after a short delay by presentation of a second target image, *B*. After another short delay they received a test comprising a simultaneous presentation of both *A* and *B*. Relative recency would be evident when participants spent more time looking at the first-presented image, A. As explained above, SOP can explain this effect in terms of *self-generated priming*: sample presentations of *A* and *B* will put elements of both these images into A1, from where they rapidly decay into A2, and then only slowly into the resting, inactive state I. If an image’s elements are predominantly in A2 on test, as they will be shortly after that image is presented, few will be available to enter A1 and it will support only weak responding. Conversely, the more time that passes, the more elements will have had time to return to the inactive state, and be available to enter A1 again at test. As elements of the first-presented image, *A*, will have had more time to decay from A2 to the inactive state than those of the second-presented image *B*, they will be more susceptible to A1 recruitment at test, and support a stronger visual orienting response.

The two other relative recency conditions examined a further prediction of the SOP account, that this self-generated priming-mediated recency effect can be modulated by the second, retrieval-priming process. Specifically, during sample presentations the various target images may also become associated with the surrounding context, which acquires the ability to prime those images’ elements directly into the A2 state, and this effect is superimposed on the relative recency effect (cf. Tam et al., 2015). We manipulated this process, by accompanying each A and B image with its own *context* stimulus, *x* or *y* (a trial-unique image of a colorful maritime flag). Specifically, image *A* was accompanied by context *x*, and image *B* by context *y*. At test the compound presentation of *AB* was accompanied either by *x* or by *y*. This manipulation should moderate the relative recency effect in different ways in the two conditions. First, during the sample phases *x* should become uniquely able to prime image *A*, and *y* image *B*. During the test when *A* and *B* are presented, the self-generated priming mechanism of relative recency described above will ensure that elements of A, having had more time to decay from the A2 state than elements of the second-presented B, will evoke stronger responding. However, if *x* is also present (the RR − OIP condition), it will selectively prime elements of the first-presented *A* into A2, counteracting the advantage of its less recent presentation, and hence dampening the relative recency effect. Conversely, if *y* is present (in the RR + OIP condition) it will prime elements of the second-presented *B*, adding to its already higher A2 activity, with the result that the recency effect should be enhanced. Thus, we predicted that the relative recency effect should be greater in magnitude in the *yAB* test of the RR + OIP condition than the *xAB* test of the RR − OIP condition.

We also manipulated the intervals between the various image presentations. According to SOP the self-generated priming mechanism of recency is intrinsically time-dependent, for two reasons. First, it depends on the difference in activation state of *A* and *B* on test that arises because one is presented *after* the other. Clearly, the longer the delay between the sample phases, the greater this difference will be. Conversely, the effect of this difference is necessarily transient, as it depends on some stimulus elements not having had time to return to their inactive states. Thus, the interval between the second sample phase and the test is also critical in determining the size of the recency effect: If the test occurs long enough after both sample presentations, both *A* and *B*’s elements will have fully returned from A2 to the inactive state, and be able to recruit A1 activity on test with equal vigor. As it is an empirical issue to determine the ideal parameters for obtaining a relative recency effect, in the present study we comanipulated the sample—sample and the second sample—test intervals, such that in each of the three conditions described above these delays were both either 0.5, 1, or 2s.

### Method

#### Participants

There were 26 female and 9 male staff and students from the University of Nottingham who participated. Their mean age was 22.9 years (range = 18–36) and they received course credit or an inconvenience allowance of £5. All reported having normal or corrected-to-normal vision; six wore glasses and two contact lenses, which they kept on during the experiment. Informed consent was obtained in accordance with the University of Nottingham School of Psychology Ethics Committee.

#### Apparatus

Eye movements were recorded with a Tobii TX300 eye tracker (Tobii Technology, Stockholm, Sweden) sampling gaze from both eyes at 300 Hz. Participants sat in front of a thin-film-transistor, with a nominally 23-in. diagonal display (51 cm wide × 28.5 cm high). Its resolution was 1920 × 1080 pixels and its refresh rate was 60 Hz. A chin rest was placed 50–60 cm from the screen to reduce participants’ head movements. A Windows 8.01 Pro Dell machine running PsychoPy (1.82.02; Peirce et al., 2019) and Tobii Studio (Version 3.4.8.1348) was used for stimulus presentation and data collection.

#### Stimuli

Two sets of stimuli were used. One set consisted of 564 images selected from the combined Bank of Standardized Stimuli (BOSS 2010 Brodeur et al.; Brodeur, Dionne-Dostie, Montreuil, & Lepage, 2010; Brodeur, Guérard, & Bouras, 2014), and matched in luminance to reduce the effects of low-level visual features using the SHINE toolbox (Willenbockel et al., 2010). The other set comprised 43 colored international maritime signal flags, either downloaded from Wikipedia (CC0, public domain license) or created using graphical software. There were three types of stimulus: *target* stimuli (*A* and *B* in Table 1), *context* stimuli (*x* and *y* in Table 1) and *catch trial* stimuli depicting items of clothing (these being used to maintain participant attention; see below). Thirty-five images from the BOSS stimulus set were allocated to the *catch* trial stimuli, and the remainder were used for the *target* stimuli (*A* and *B*; see Table 1). The maritime flags served as the *context* stimuli. Stimuli were resized to 350 × 350 pixels, and on each trial the identity of each specific stimulus type (*A*, *B*, *x*, *y*) was determined by sampling randomly from the corresponding image pool. Target, catch trial and context stimuli were sampled without replacement; however, because number of context stimuli was relatively limited, when only one flag remained the entire context stimulus set was made available again for sampling, and this process was repeated as many times as required. The stimulus allocation process was applied independently to each participant, thus randomizing stimulus identity across the different task conditions.[Table-anchor tbl1]

The stimuli could be presented in one of four locations: top left, top right, bottom left, and bottom right, equidistantly from the center of the screen. The central coordinates of these positions on the horizontal and vertical axes (in pixels) relative to the center of the screen (0,0) were: top left (−275, 275); top right (275, 275); bottom left (−275, −275), and bottom right (275, −275).

#### Procedure

Before the experiment began, participants completed a short eye-tracking calibration routine to ensure gaze was being tracked, and then they were guided through the instructions. Participants were told not to memorize specific stimuli but to press the space bar every time they saw an item of clothing (*catch* trials).

There were four types of trial: three types of experimental trial, *AB*, *xAB*, *yAB*, and *catch* trials. The *experimental* trials, 36 of each type, all followed the same format of two, sequential 3-s sample phases, each comprising a presentation of one of the target stimuli (i.e., *A* then *B*), followed by a 3-s test phase in which both of the target stimuli from the sample phases were represented (*AB*). In addition, on *xAB* and *yAB* trials *context* stimulus *x* accompanied *A* in the sample phase, and context stimulus *y* accompanied *B*. In the test phase *x* was present on *xAB* trials, and *y* on *yAB* trials (see Table 1). For equal numbers of each of these three trial types the *delay* between the two sample phases, and also between the second sample and the test phase, was 0.5, 1, or 2 s (the sample-sample and sample two-test delays were always identical on a given trial).

The three trial types *A* appeared an equal number of times in each of the four possible locations in the first sample phase; in the second sample phase B was randomly placed in one of the positions on the opposite side of the screen (e.g., *A* top left → *B* top right or bottom right, etc.). At test *A* and *B* maintained their sample-phase positions. On *xAB* and *yAB* trials the location of context stimuli *x* and *y* was randomly assigned to one of the unoccupied locations.

In addition, participants received 18 *catch* trials, to maintain attention during the task. These were of the same form as the experimental trials, with nine of each trial type, three of each set of nine with a delay of 0.5-s, three of 1 s, and three of 2 s. In all cases either *A* or *B* was randomly replaced by one of the catch trial stimuli.

Participants received these trial types in a semirandom order, separated by a 1-s intertrial interval. Participants were given the opportunity to take a break for as long as they wished, after the 22nd, 43rd, 64th, 85th, and 106th trial. The sessions lasted between 30 to 45 min.

#### Data treatment

Eye-tracking data were processed and dwell times calculated in R Studio using the rhdf5 (Fischer, Smith, & Pau, 2019) packages. Initially data from catch trials were removed from the data set, as was the data from all experimental trials on which the participant pressed the space bar (i.e., mistakenly treating it as a catch trial; this constituted less than 2% of the total). For the remaining data, gaze location for left and right eyes was averaged for each sample, producing a single gaze location in the x- and y-axes. On average, participants maintained attention to the experimental stimuli; the mean percentage of time during which gaze could not be tracked (either because it was not detected by the software, or participants were not looking at the screen) was low, at between 12 and 13%.

Two areas of interest (AOIs) were defined for each test trial, which were coextensive with the positions in which target stimuli *A* and *B* were presented, and active only during phases in which those stimuli were presented. Data from the context stimuli are not presented. Dwell times within these AOIs were computed in six, 0.5-s bins beginning at stimulus onset; however, because initiating a saccade can take of the order of 200 ms, data from the first bin were excluded (cf. Carpenter, 1988). Conversely, visual inspection of the data revealed that most differences had dissipated by bin 4. Thus, the data presented below are average values for bins 2 and 3, corresponding to the temporal window between 0.5 and 1.5 s after stimulus onset. These values, expressed as percentage of time per AOI (*gaze scores*), were computed for the first and second sample phases (averaged over both target stimuli in each sample phase), and for the first-presented *A* and the second-presented *B* in the test phase, separately for each delay in each of the three types of trial. In addition, these test values for *A* and *B* were expressed as *discrimination ratios* of form (*a−b*)/(*a + b*), where *a* is the test gaze score for *A*, and *b* the corresponding score for *B*. A value of 0 indicates no preference, while a value of 1 means the participant looked only at the first-presented *A*. Thus, the higher the score, the stronger the relative recency effect.

Data were analyzed using repeated measures analysis of variance (ANOVA); reliable interactions were explored with simple main effects using the pooled error term or, where appropriate, *t* tests using Holm-Bonferroni *p* values to control for the family wise error rate. Statistical analyses were two-tailed with α = .05. Partial eta squared (η_p_^2^) was used to represent main effect and interaction effect sizes. Standardized 90% confidence intervals (ICs) for η_p_^2^ were computed using the methods described by Kelley (2007) and used his MBESS package. Inferential statistical analysis was performed in SPSS and JASP.

### Results and Discussion

The mean gaze scores for each trial type at each delay during the preexposure phase are shown in Table 2, for the first- and second-presented samples, *A* and *B*, respectively. First, it is evident that these scores were higher for RR trials than either RR − OIP or RR + OIP trials, with mean values, averaged across delay, of 68.89, 52.95, and 53.17, respectively. This is to be expected, as the contextual stimuli appeared in the RR − OIP and RR + OIP sample phases, but never in the RR sample phases. To the extent that participants looked at the contextual cues, this would reduce the time available for them to look at *A* and *B*. This impression was confirmed by an ANOVA with condition (RR, RR − OIP, RR + OIP), delay (0.5, 1, 2), and sample (*A*, *B*) as factors. This revealed a reliable effect of condition, *F*(2, 68) = 157.21, *MSe* = 111.61, *p* < .001, η_p_^2^ = .82, CI [.751, .857] and the RR condition was reliably higher than both RR − OIP and RR + OIP, *p*s < .001; the latter two conditions did not differ, *p* = .84. The ANOVA also revealed a main effect of sample, *F*(1, 34) = 13.70, *MSe* = 31.00, *p* < .001, η_p_^2^ = .29, CI [.089, .455]; nothing else was significant, largest *F*(2, 68) = 2.72, *MSe* = 31.08, *p* = .073. The effect of sample reflected the fact that on average gaze scores for the first-presented A were marginally lower than for the second-presented B, with means of 57.52 and 59.16, respectively. This probably arose because sample A could appear in any of the four locations, but if sample A appeared on the left, for example, sample B would necessarily appear on the right. This reduction in uncertainty in where the second sample would appear could reduce the time required to initiate a saccade in that direction, and marginally increase looking time. However, while the effect was statistically reliable, it was small (less than .01 s) and so was unlikely to have influenced the effects of interest.[Table-anchor tbl2]

**Figure 1 fig1:**
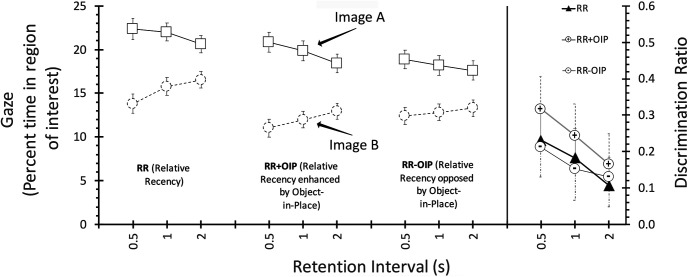
The left panel depicts mean gaze scores for the first- and second-presented images, A and B, respectively, in the test phase of Experiment 1; values are presented separately for each delay in each trial type. The right panel represents the discrimination ratios derived from these values. For more information see text.

The test data are presented in Figure 1. In the left panels, which depict gaze scores for the A and B at test, it is evident that participants in the RR condition spent more time looking at the first-presented image, *A*—the relative recency effect; this effect appears larger in the RR + OIP condition, but similar to RR in the RR − OIP condition. In all conditions the difference reduced as delay increased. These impressions were supported by the results of an ANOVA, with condition (RR, RR − OIP, RR + OIP), delay (0.5, 1, 2), and image (*A*, *B*) as factors. This revealed main effects of condition, *F*(2, 68) = 48.17, *MSe* = 47.05, *p* < .001, η_p_^2^ = .59, CI [.477, .666], image, *F*(1, 34) = 63.05, *MSe* = 418.224, *p* < .001, η_p_^2^ = .65, CI [.467, .742], a reliable interaction between image and delay, *F*(2, 68) = 6.41, *MSe* = 109.76, *p* = .003, η_p_^2^ = .16, CI [.036, .274]; nothing else was significant, largest *F*(2, 68) = 2.86, *MSe* = 103.26, *p* = .064. Exploration of the Image × Delay interaction confirmed that the A/B discrimination was reliable at all three delays, *p*s < .001.[Fig-anchor fig1]

The right panel shows the corresponding discrimination ratios, computed from the gaze data described above. Discrimination between the first-and second-presented images was greater in the RR + OIP condition than the RR and RR − OIP conditions; again, the discriminations reduced as delay increased. ANOVA with condition and delay as factors revealed main effects of both condition and delay, *F*(2, 68) = 3.97, *MSe* = .012, *p* = .023, η_p_^2^ = .11, CI [.008, .211] and *F*(2, 68) = 7.81, *MSe* = .012, *p* = .001, η_p_^2^ = .19, CI [.055, .304], respectively; the interaction was not significant, *F* < 1. Ratios for RR + OIP differed reliably from the RR − OIP, *p* = .037, but not from the RR condition, *p* = .052; the RR and RR − OIP conditions did not differ, *p* = .76. It should be noted, however, that direct comparison between the RR condition and the others must be treated with caution, as the RR + OIP and RR − OIP treatments included a contextual stimulus during both training and test, whereas the RR condition did not.

### Discussion

A relative recency effect was obtained in this study: dwell times were greater for the first presented stimulus *A* than for the second-presented *B*, and this was true in all three conditions. This can be interpreted within the terms of SOP as an effect of *self-generated* priming: the elements of stimulus *A*, which was presented temporally further from the test than *B*, would be more likely to have decayed from A2 to the inactive state at the point of test, making them more available for recruitment to A1, and hence more able to elicit a strong visual orienting response.

We also found evidence that this effect was reliably influenced by the identity of the contextual cue that appeared at test, being reliably smaller in the RR − OIP condition in which *x* was present at test than in the RR + OIP condition where *y* was present. SOP would explain this as an instance of *retrieval-generated* priming superimposed on the recency effect. During the sample phase, *x–>A* and *y–>B* associations would form, so that on *xAB* test trials *x* would prime *A*’s elements directly into A2. Thus, on *xAB* trials *A*’s tendency to elicit a stronger visual orienting response would be offset by *x* priming more of its elements into the A2 state. Conversely, on *yAB* trials *B*’s visual orienting response, already limited because of its elements being more in A2 than those of the less recently presented *A*, would be reduced still further by *y* priming more of *B*’s elements into A2. Thus, retrieval-generated priming would reduce the recency effect on *xAB* trials and increase it on *yAB* trials. While this experiment could not provide evidence that recency was reliably reduced in the RR − OIP condition, it was clearly greater in the RR + OIP condition, which is consistent with this analysis.

Finally, we also observed an effect of delay: the recency effect was reliably greater at the shorter delays in all three conditions, although present at all of them. It is unclear what Wagner’s model would predict about the effect of delay in this instance because of the covariation of intersample interval with sample-test interval. On the one hand the longer the delay between the two samples, the greater the difference in A2 activity between the first- and second-presented images at test, and hence the *better* the recency effect. Conversely, the longer the interval between the second sample and the test, the more time elements of the second sample have to return to the inactive state, and hence the *worse* the recency. The results could suggest that the second of these factors is predominating, resulting in the poorer performance at longer delays. Here we should note that these results contrast with those reported in a recent study by Barker, Evuarherhe, and Warburton (2019). These authors conducted a series of experiments in which rats were exposed to a sequence of four different stimuli, S1, S2, S3 and S4; in their Experiment 1 both the intersample interval and the interval between the final sample and the test were either 5-min or 60-min, in a formal parallel of our procedure in the present study. They were then tested with either S1 and S4, or S2 and S3. They reasoned that any account of recency based on temporal decay, such as that presented here, should predict the discrimination between S1 and S4 to be more robust than that between S2 and S3. However, in contrast to our results, Barker et al. failed to detect any influence of intersample interval on the magnitude of relative recency. We will discuss potential reasons for this discrepancy below.

## Experiment 2

In Experiment 1 we demonstrated relative recency in this eye-tracking paradigm, and showed that this effect may be moderated by manipulation of the contextual cues accompanying the sample and test phases. The recency effect may be interpreted as providing evidence of differential self-generated priming, while the moderation of this effect by the contextual cues is indirect evidence of retrieval-generated priming. The first aim of Experiment 2 was to replicate the recency effect in this preparation; the second was to look for more direct evidence of retrieval-generated priming in the same procedure, by examining whether it could bias gaze under conditions in which self-generated priming is equated.

In Experiment 2 we modified the procedure used in Experiment 1 so that the stimuli were presented in pairs rather than individually, and all conditions were tested under identical conditions, with four-stimulus arrays (see Table 1). Thus, in the relative recency (RR) condition participants were presented with a sequence of stimulus pairs, a sample presentation of *AB*, followed by a second sample of *CD*. In the test that followed they received an array of *A*, *B*, *C*, and *D*; more gaze at the first-presented pair of stimuli, *AB*, would constitute a relative recency effect (see Table 1). The *Object-in-Place* condition (OIP) was designed to find evidence of retrieval-generated priming. Rather than using explicit context cues to increase priming effects at test, we relied on the supposition that both the absolute position of a stimulus on the screen, and its position relative to other stimuli on the screen, could result in associations predicting its presence in a specific location, analogous to the locations in the arena of a rodent object recognition task. This means presenting an image at test in a different location should disrupt the ability of these associations to prime its elements into A2, and increase its visual orienting response. Thus, in the OIP condition participants received two identical sample phases comprising presentation of the four-stimulus array *ABCD*. At test they were presented with an array that was identical except that two of the stimuli exchanged position—for example, ***BA***CD. More gaze at those stimuli whose locations had switched was taken as evidence of retrieval-generated priming; moreover, as all the stimuli in the array had been experienced equally recently, there should be no possibility of differential self-generated priming of the different components of the test array.

A further aim was to replicate the moderation of the recency effect by retrieval-generated priming that was observed in Experiment 1. To achieve this, we included two further conditions, RR + OIP and RR − OIP, which comprised the same sample phases as condition RR—a sample presentation of *A* and *B* followed by presentation of *C* and *D*. At test, members of one of these stimulus pairs switched location, such that the test array comprised **BA**CD (RR + OIP) or *AB***DC** (RR − OIP). This should moderate the size of the relative recency effect, as it did in Experiment 1. Specifically, when members of the first-presented pair, *AB*, switch location (***BA***CD; RR + OIP), the resultant loss of retrieval-generated priming should *increase* the recency effect: this first-presented stimulus pair would normally be inspected more because its elements have had more time to decay from A2, and this would be enhanced by selective loss of retrieval-priming induced by *A* and *B* changing places. Conversely, the recency effect should *decrease* when members of the second-presented pair *CD* switch location (*AB***DC;** RR − OIP): here, although *CD*’s elements will have had less time to decay from A2 than those of *AB*, this will be counteracted by their being primed less into A2 than elements of *AB*.

We also compared the effect of having a 1 or 10-s retention interval between the second sample and the test in all four conditions; the sample—sample interval remained fixed at 1s. We predicted that the recency effect in condition RR, being dependent on time-sensitive self-generated priming, should be greater after 1 s than after 10 s, by which time elements of both stimulus pairs should have largely returned to the inactive state. The OIP effect, on the other hand, should not be sensitive to retention-interval duration, as it depends on associations formed during preexposure producing retrieval-generated priming at test, and these associations should not diminish substantially over time. Related predictions can be made for the other two conditions, where we predict that retrieval-generated priming should be disrupted for the *AB* images in the (RR + OIP), and so increase the size of the recency effect; conversely, disrupting retrieval-generated priming in the second pair of images, *CD*, in the (RR − OIP) condition, should reduce recency. Moreover, as they depend on retrieval-generated priming, these effects should be present at *both* retention intervals; thus, if the relative recency effect has completely dissipated after the 10-s delay, then we might expect the increased orienting to *AB* to persist at 10-s in the (RR + OIP) condition, but be reversed in the (RR − OIP) condition.

### Method

Unspecified details were identical to those of Experiment 1.

#### Participants

There were 35 female and 22 male students or staff at the University of Nottingham who participated. Their mean age was 37.8 years (range = 18–81). One of the purposes of this study was as a pilot to ensure the task was suitable for elderly participants, hence the broad age range; however, we did not detect any notable effects of age on performance, and so this factor was not included in the analyses. Participants were recruited through opportunity sample from a public-engagement event. All participants completed the experiment and reported normal or corrected-to-normal vision; 27 wore glasses and five contact lenses during the experiment. Data from one participant were excluded because of apparatus failure, leaving 57 participants.

#### Stimuli and apparatus

The flag stimuli were not used in the present study.

#### Procedure

There were no catch trials in the present experiment, and participants were simply instructed to pay attention to the stimuli presented on the screen. There were four different conditions, all comprising two sample phases, separated by a 1-s interval, and a test phase. In the RR, RR + OIP, and RR − OIP conditions the sample phases each comprised a pair of stimuli, *AB* followed by *CD*; on half the trials of each type *AB* was presented in the upper half of the screen, and *CD* on the lower half of the screen, and for the remaining trials the reverse. In the OIP condition the same array of four stimuli *ABCD* was presented in both sample phases. The test phase of each trial comprised the four stimuli presented in the sample phases. For condition RR each stimulus appeared in the same position at test as in the sample phases; for half the test trials in each of the remaining conditions (OIP, RR − OIP, RR + OIP) the position of the two stimuli in the top *or* bottom half of the screen were swapped. On RR + OIP trials it was always members of the *first*-presented pair *AB* that were switched at test, while on RR − OIP trials it was always members of the *second*-presented pair *CD*; for half the OIP trials the top *AB* pair of images was reversed at test, and for the remaining trials the bottom pair, *CD*. Finally, half the trials in each condition had a 1-s interval between the second sample and the test phase, and the remainder a 10-s interval; this factor was fully counterbalanced across the position (top or bottom) of the switched stimulus pair. There were 16 trials in each of the four conditions, and participants were able to take a break if they wished after Trials 15, 31, and 47.

#### Calculations and data treatment

Data from the sample phases, averaged over all the images presented, were computed separately for all images present during each of the first and second sample phases for each trial type and retention interval. Data from the test phase were calculated separately for the stimulus pairs at the top and bottom of the screen. For the OIP task these stimulus pairs were categorized as having switched position (*a*) or not (*b*), while for the RR, RR + OIP, and RR − OIP conditions they were categorized according to whether they had appeared in the first (*a*) or second (*b*) sample phase. Thus, in the OIP and RR conditions we predicted higher gaze scores for *a* pairs than *b* pairs because of object-in-place and relative recency effects, respectively, while in the RR + OIP and RR − OIP conditions we could evaluate the extent to which this relative recency effect was modulated by the position switch of the first- and second-presented sample pairs, respectively. In all other respects data were treated exactly the same as in Experiment 1.

### Results

The mean gaze scores per AOI for each trial type at each retention interval during each of the two sample phases are shown in Table 3. Scores were lower on the OIP trials than in the other conditions, presumably because OIP samples contained four images, and hence four AOIs, whereas the remaining trial types comprised only two, so there were fewer images to look at. Also, as in Experiment 1, there was a small but consistent tendency for participants to have higher gaze scores for the second sample than the first. ANOVA with trial type (OIP, RR, RR + OIP, RR − OIP), retention interval (1, 10) and sample (first, second) as factors revealed reliable main effects of trial type, *F*(3, 168) = 1370.939, *MSe* = 15.65, *p* < .001, η_p_^2^ = 96, CI [.951, .966], sample, *F*(1, 56) = 17.12, *MSe* = 5.01, *p* < .001, η_p_^2^ = .23, CI [.087, .374], and an interaction between these two factors, *F*(3, 168) = 2.96, *MSe* = 3.51, *p* = .034, η_p_^2^ = .05, CI [.002, .100]; there was a reliable effect of sample in the RR, RR + OIP, and RR − OIP conditions, *p*s < .036, but not OIP, *p* = .9. As in Experiment 1, these differences were small, and probably simply the result of the information that the first sample image gave on where the second one was to be situated. In the OIP condition four images were presented in both samples, while in the RR conditions, if the first sample images were at the top of the screen, the second would be at the bottom (and vice versa), creating the possibility of prospective looking before the samples actually appeared in the latter condition.[Table-anchor tbl3]

**Figure 2 fig2:**
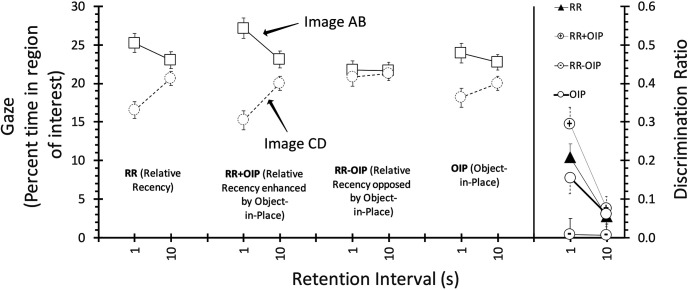
The left panel depicts mean gaze for image pairs, AB and CD, respectively, in the test phase of Experiment 2 (see Table 1); values are presented separately for each retention in each condition. The right panel represents the discrimination ratios derived from these values. For more information see text.

The test data are presented in Figure 2. The left panel shows gaze scores for the *AB* and *CD* pairs of images (see Table 1), where *AB* were the images that exchanged location in the OIP condition, and were the first-presented pair in the RR, RR + OIP, and RR − OIP conditions. In general participants looked more at *AB* than *CD* in all conditions, and especially at the shorter retention interval, and this effect was more marked in the RR + OIP conditions than the RR − OIP condition. ANOVA with condition (OIP, RR, RR + OIP, RR − OIP), retention interval (1, 10) and image pair (AB, CD) as factors revealed a reliable three-way interaction, *F*(3, 168) = 3.49, *MSe* = 54.76, *p* = .017, η_p_^2^ = .06, CI [.010, .111]; the main effects of retention interval and image pair were also significant, *F*(1, 56) = 8.17, *MSe* = 6.18, *p* = .006, η_p_^2^ = .13, CI [.022, .262] and *F*(1, 56) = 53.73, *MSe* = 84.70, *p* < .001, η_p_^2^ = .49, CI [.327, .599], respectively, as were the interactions between image pair and both condition, *F*(3, 168) = 7.74, *MSe* = 61.53, *p* < .001, η_p_^2^ = .12, CI [.045, .189], and retention interval, *F*(1, 56) = 17.67, *MSe* = 70.39, *p* < .001, η_p_^2^ = .24, CI [.092, .380]. Nothing else was significant, largest *F*(3, 168) = 1.33, *MSe* = 4.74, *p* = .27.[Fig-anchor fig2]

The three-way interaction was analyzed further with separate ANOVAs for each condition, with retention interval and image pair as factors. For the OIP condition there was a main effect of image pair, *F*(1, 56) = 15.15, *MSe* = 68.03, *p* < .001, η_p_^2^ = .21, CI [.072, .353], but no effect or interaction involving retention interval, largest *F*(1, 56) = 2.05, *MSe* = 764.479, *p* = .16. However, for both the RR and RR + OIP conditions there was a reliable interaction between image pair and retention interval, *F*(1, 56) = 12.40, *MSe* = 46.29, *p* = .001, η_p_^2^ = .18, CI [.051, .321] and *F*(1, 56) = 16.38, *MSe* = 66.59, *p* < .001, η_p_^2^ = .23, CI [.084, .369] for RR and RR + OIP, respectively; however, while in the RR + OIP task the effect of image pair was reliable at both retention intervals, *p* < .001 and *p* = .049 for the 1-s and 102 retention intervals, respectively, in the RR task the effect of image pair was reliable at the short retention interval, *p* < .001, but not the long, *p* = .07. Finally, in the RR − OIP condition nothing was significant, *F*s < 1. Thus, the OIP condition demonstrated an object-in-place effect that was unaffected by retention interval, while the recency effect was eliminated at the longer retention interval. In the RR + OIP condition, where the two effects were operating together, relative recency was numerically smaller but still reliable at the longer retention interval. In contrast, in the RR − OIP condition, where the recency and object-in-place effects were opposing each other, there was no sign of a recency effect at either interval.

The right panel of Figure 2 shows the discrimination ratios. As with the gaze scores, the ratios seemed to differ at the short but not the long retention interval. ANOVA with condition and retention interval as factors revealed a main effect of both factors, *F*(3, 168) = 7.77, *MSe* = 017, *p* < .001, η_p_^2^ = .12, CI [.045, .190] and *F*(1, 56) = 21.09, *MSe* = .02, *p* < .001, η_p_^2^ = .27, CI [.118, .411], and an interaction between them, *F*(3, 168) = 3.18, *MSe* = .016, *p* = .025, η_p_^2^ = .05, CI [.004, .105]; there was a reliable effect of condition at the short but not at the long retention interval, *p* < .001 and *p* = .47, respectively. At the short retention interval, the ratio for RR + OIP was greater than for RR − OIP, *p* < .001, and also OIP condition, *p* = .035; in addition, the RR − OIP ratios were lower than those for the RR condition, *p* = .003. There were no differences at the long retention interval. In addition, there was an effect of retention interval for the RR and RR + OIP conditions, *p*s < .001, but not for the OIP or RR − OIP conditions (*p* = .12 and .6, respectively).

### Discussion

The first aim of Experiment 2 was to replicate the relative recency effect observed in Experiment 1, and to demonstrate the object-in-place effect. The most significant aim of Experiment 2 was to test the prediction that the recency effect should be attenuated with an increase in the retention interval between the second sample phase and the test, but that the object-in-place effect should not—a prediction derivable from Wagner’s SOP model. Thus, in the RR condition participants experienced a presentation of *AB*, followed by another of *CD*, and a test of *ABCD*. Recency was demonstrated, in that participants spend more time at test looking at the first-presented image pair *AB*; however, this effect was present only at the shorter retention interval. In the OIP condition participants experienced two identical sample presentations of two stimulus pairs, *AB* and *CD*; however, at test, one of these pairs (e.g., *AB*) exchanged position. We found, as we predicted, that participants would spend more time looking at the displaced *AB* pair than *CD*—and this effect was unaffected by the length of the retention interval. This pattern of results falls directly out of our SOP analysis: according to this account, the relative recency effect arises because, by the test, elements of the first-presented image pair *AB* will have had more time to return from A2 to the inactive state than *CD* and, thus, be more ready to enter A1 and elicit a strong visual orienting response. However, at the longer, 10-s retention interval, this self-generated priming effect would have dissipated, meaning elements of both image pairs would have had sufficient time to decay completely from A2 to I, so *AB*’s advantage would be diminished, and recency abolished. In contrast, the object-in-place effect relies on retrieval-generated priming, that depends on associations formed between the images and the cues that surround them; as these associations are permanent, we would not expect this effect to be temporally transient—and indeed the OIP effect was equally robust at both the short and the long retention interval.

Although the effect of retention interval was not significant in the OIP condition, there did seem to be a numerical trend toward a reduction of the effect at the longer delay, this being especially evident from the ratio measure. Although such a trend could be accommodated within our theoretical analysis—either through weakening of associations over time, or a progressive change of context between the sample and test conditions as the retention interval elapses, which would attenuate the degree to which the priming associations could be retrieved (cf. Bouton, 1993)—it may also indicate a weakness in our SOP-based analysis. However, applying other variations of an associative learning model might do better in this respect. For example, McLaren, Kaye, and Mackintosh (1989; see also McLaren, 1994) proposed an account of associative learning that also discriminates between direct and indirect activation of stimulus elements, albeit in a rather different manner to SOP, and that could also account for some of the effects reported here. Interestingly, it also proposes that on a learning trial associative strength initially increases, but then decays slightly before reaching a new asymptote; Thus, if tested immediately an association would appear rather stronger than after a short delay. This kind of mechanism could perhaps accommodate the marginal effect of retention interval on our OIP task.

Another consideration in the OIP task is the possibility of generalization decrement. Performance on tasks of this kind can be explained quite simply by arguing that when the target image changes location, it is perceived as a slightly different and, therefore, novel object, and so the return of the unconditioned response is not because of retrieval failure but to a new unconditioned response to an effectively novel cue. We took no explicit measures to address this possibility in the present study, although one could argue that the change in perception of the same image viewed at different positions on the screen is likely to be minimal. Nonetheless, the logical possibility remains—and some experimenters have developed techniques to address this. For example, Whitt et al. (2012) trained rats in a task in which object P was presented with stimulus X, and object Q with stimulus Y. This would allow X and Y to become, respectively, associated with P and Q, respectively. Rats were then tested with P and Q in their training locations, and in the absence of either X or Y—but in the interval between preexposure and test all animals experienced X. The authors reasoned that presentation of X before the test should be sufficient to prime elements of object X into the A2 state, resulting in a greater exploration of object Y—which is the result they observed. Critically, the conditions on test were matched for X and Y, so that it was not possible to attribute this difference to differential generalization decrement of the two objects.

The second aim of this study was to replicate the effect observed in Experiment 1, that the recency effect could be modulated by retrieval-generated priming effects. In the RR + OIP and RR − OIP conditions, participants also experienced presentations of *AB* followed by *CD*, and then a test with *ABCD*—but in addition components of either the first-presented or second-presented image pairs exchanged position at test (***BA***CD or *AB***DC** in the RR + OIP and RR − OIP conditions, respectively). Changing the positions of the stimuli should disrupt the potential for retrieval-generated priming and increase visual orienting to that stimulus pair. Thus, when AB is switched in RR + OIP, the recency effect should be enhanced, while if CD is switched, in RR − OIP, it should be diminished. This was what we observed: As in Experiment 1, there was a greater recency effect in the RR + OIP than the RR − OIP condition. We note that the recency effect in the RR + OIP and RR groups differed in its sensitivity to retention interval, with recency being abolished at the longer interval in the RR condition, but not in the RR + OIP condition. This also accords with our analysis. In the RR condition recency depends entirely on self-generated priming, which is temporally transient; in the RR + OIP condition, on the other hand, a component of the recency effect is because of retrieval-generated priming that is not time-dependent, and so the effect was maintained at the longer retention interval.

## General Discussion

In two eye-tracking experiments we found that human participants given sequential exposure to two sets of images tended to inspect the first-presented image more than the second in a subsequent test—the relative recency effect. Although there is a substantial literature reporting this effect in rodents (e.g., Mitchell & Laiacona, 1998), we believe this to be the first demonstration of this effect using a behavioral measure in human participants. We also found that, after exposure to an array of images, in a subsequent test, participants spent more time inspecting images in the array that had changed location at test compared with those that had not. This parallel of the object-in-place task used in rodents (Dix & Aggleton, 1999) has previously been used in a handful of human studies (Hannula et al., 2007; Mahoney et al., 2018; Richmond & Nelson, 2009; Ryan & Cohen, 2004; Yeung et al., 2019), but their results are mixed. For example, Mahoney et al. (2018) allowed participants to study pictures of various faces superimposed on different scenes. At test they saw one of the scenes with three faces, one of which had appeared with that scene during the study phase. In contrast to our findings, they found participants spent more time looking at the matching faces, that *had* appeared on that scene in the preexposure phase (see also Hannula et al., 2007). In contrast, Ryan and Cohen (2004) showed participants pairs of scenes, where the second was either identical to the first (match) or differed through deletion, addition, or shift of an item in the scene. They found that participants spent more time viewing the second scenes of a pair when they did *not* match the first scene, compared with when they were identical (see also Yeung et al., 2019). Given the many procedural differences between these two sets of studies, it is unclear what is responsible for the discrepancy in their results; but our results accord with those of Ryan and Cohen (2004) and the plethora of parallel studies in the animal literature, showing greater tendency to inspect the misplaced images.

In our studies the relative recency effect was very transient, as predicted by the SOP model; but given this brevity, one might doubt the extent to which it could be involved in what we understand as normal recognition memory, which is typically temporally robust. In fact, in some circumstances recency effects of the type reported here do seem to persist over longer intervals than the self-generated priming mechanism proposed by the SOP account would imply (cf. Mitchell & Laiacona, 1998). Another challenge to this version of the account comes from the study mentioned earlier by Barker et al. (2019), in which rats were no better at discriminating between the first and fourth items of a four-item sequence than the second and third. If the temporal decay of secondary A2 activation is as rapid as our results suggest, then we would certainly expect to see differences in the Barker study, with better discrimination of the more temporally separated items. Given the large number of procedural differences between the studies, not least the species, it is difficult to know what could be responsible for the difference in results—but one possibility is the duration of sample exposure. In our study exposures were, at 3 s, very brief, whereas in the Barker et al. study the rats had 4 min to explore the objects during the preexposure phase. This could have a number of effects. First, it is possible that this longer exposure time could have fostered formation of associations, both between the object and the surrounding context, and also among elements of the object *itself*. Both these types of association would result in retrieval priming at test: the context → object associations would allow experiencing the surrounding context to prime elements of the target object, while any within-compound associations forming among components of the target object would allow experiencing one component of the target object to prime others. Critically, this priming would occur for both the objects at test, meaning that if these effects were sufficiently robust they could swamp the more subtle differences in self-generated priming, allowing the SOP account to explain Barker et al.’s results. However, in the absence of further experimental work this suggestion must remain speculative.

This leads onto the further question of whether, given the apparent transience of the effect, our task should be regarded as a measure of recognition memory at all. We have defined recognition as the ability to discriminate between items that have been previously encountered from those that have not. In terms of this definition our findings safely qualify—but is this sufficient? For example, in the human memory literature there is a distinction between recognition memory and what is termed repetition priming, “*a long-term change in the identification, detection or production of an item as a result of prior exposure to that item*” (Berry, Shanks, Speekenbrink, & Henson, 2012), and there are many models that assume that these phenomena should be regarded as products of independent memory systems, with recognition being a type of explicit declarative memory, and repetition priming an instance of implicit, nondeclarative memory (e.g., Squire, 1994). In these terms one could argue that our task is more likely to tap a transient repetition priming effect than “true” recognition memory. However, here again a consideration of the preexposure time used in our studies is instructive. For example, it has been reported that, in the visual paired comparison task, the magnitude of the preference for gazing at the novel object was not only directly related to exposure time, but with a preexposure duration of 30 s persisted for a month (Richmond, Sowerby, Colombo, & Hayne, 2004). As our exposure times were a tenth of this value, this could indeed be a critical factor—and suggests that if we had used longer exposure times, more stable effects would have been obtained. Moreover, these same authors argued that VPC should be regarded as a measure of declarative memory, noting both that people with amnesia and participants with hippocampal damage—both which are assumed to have deficits in declarative memory—show deficits on the VPC task, and that the effects of study time and retention interval on VPC performance bear a “striking similarity” to the effects of these same variables on declarative memory (Richmond et al., 2004). Perhaps a more parsimonious view is, therefore, that both the type of repetition priming seen in the VPC task and recognition memory are actually different measures of the same memory trace, and that both may be explained in terms of the same underlying process (e.g., Berry et al., 2012; Hintzman, 1988). Understood in those terms, we would argue that performance on our task simply represents one of several measures of this underlying memory trace strength that is also responsible for performance on more orthodox recognition memory tasks.

We also demonstrated that the object-in-place and recency effects can interact. For example, in Experiment 1 participants viewed *A* accompanied by contextual cue *x*, and *B* by contextual cue *y*; we found that the recency effect was smaller if *x* was present at test, compared with when *y* was present. If presenting *y* at test may be thought of as producing a mismatch for *A*, this should produce an object-in-place effect, enhancing inspection of *A*, while presenting *x* at test would do the opposite—so this can explain our results. A similar finding was reported in Experiment 2, where participants experienced presentation of *AB* followed by *CD*, and were then tested with an array of all four images—but in the RR + OIP condition *A* and *B* exchanged position, whereas in the RR − OIP condition *C* and *D* exchanged position. A parallel effect was found: switching *A* and *B* would create a mismatch and increase viewing of this first-presented image pair, thus enhancing the recency effect, while switching *C* and *D* would do the opposite. In fact a complementary effect has been reported in rodents by Tam et al. (2015), while investigating the influence of recency on performance on a commonly used variant of the object-in-place task in which animals are first exposed to object *A* in context *x*, and then object *B* in context *y*, before being tested with *A* and *B* in either *x* or *y*. Animals typically explore *B* more in *x*, and *A* more in *y*, demonstrating memory for where these objects were presented in preexposure, and this is usually taken as evidence of an object-in-place effect. However, Tam et al. pointed out that recency could interact with performance on this task: this should produce an increased tendency to explore the first-presented object, which would boost the object-in-place effect when animals are tested in *y*, compared with when they are tested in *x*. This is precisely what they observed, and so they concluded that care should be taken interpreting performance on this task variant, as the object-in-place effect is being contaminated by the superimposed recency effect— the complement to what we found in the present studies.

We have argued that our findings can be accounted for in terms of the processes of self-generated and retrieval-generated priming proposed by SOP (Wagner, 1981). As noted above, according to this account the recency effect is produced by self-generated priming, and the object-in-place effect by retrieval-generated priming. However, many of our findings could equally well be explained by any other accounts of recency and object-in-place memory that allow the effects to occur simultaneously. Moreover, there are other models of human recognition memory that rely, like ours, on a single underlying process (e.g., Berry et al., 2012), and although designed to explain for data from human studies, can account for the effects of both item repetition and cueing on performance in memory tasks and so could perhaps accommodate our results. Nonetheless, there are perhaps two respects in which the SOP analysis perhaps has an advantage over these other accounts. The first is parsimony: SOP can explain both these effects within the same theoretical framework—a framework that can also provide a comprehensive account of many associative learning phenomena. The fact that it also provides a comprehensive account of many learning effects gives it some edge over these rivals. The second is the dependence of these effects on time. SOP is quite specific about the fact that self-generated priming should be temporally transient whereas retrieval-generated priming is not. Thus, the fact that in Experiment 2 we found that, although the recency effect dissipated over the longer retention interval, the object-in-place effect in the OIP condition did not, is completely consistent with this interpretation (cf. Tam et al., 2014). Moreover, in the RR + OIP condition, in which both effects were superimposed, although the tendency to inspect *AB* was smaller after the longer retention interval, in contrast to the RR condition it was not abolished—that is consistent with our SOP analysis. However, as noted above, SOP may not be the only associative account that can offer an account of these findings; as one example, the model proposed by McLaren et al. (1989; see also McLaren, 1994) is also a contender.

Finally, one of the advantages of this eye-tracking procedure is the high level of temporal control it offers over stimulus exposure, which is in stark contrast to the spontaneous object recognition techniques in animals, in which exploration takes place over several minutes and can be greatly affected by environmental variables distracting the animals during the procedure, and affecting the degree to which they interact with the objects. Although SOP cannot make general quantitative predictions about the time-course of the various decay processes on which the model depends, it might be possible to derive some parameters specific to a procedure such as this, in which the time-course of stimulus presentations, and the intervals separating sample and test presentations can be so precisely controlled; moreover, the change in visual inspection over time can be tracked with great precision. Exploiting these special procedural features might allow us to make much more precise and quantitative predictions than has hitherto been possible, that could allow us to further discriminate SOP from its competitors.

## Figures and Tables

**Table 1 tbl1:** Design of Experiments 1 and 2

Condition	Sample 1	ISI	Sample 2	RI	Test	SGP?	RGP?	Net bias?
Experiment 1								
RR	*A*	.5, 1, 2 s	*B*	.5, 1, 2 s	*A B*	*B*	—	*A* > *B*
								
RR + OIP	*x A*		*y B*		*y A B*	*B*	*B*	*A* >> *B*
								
RR − OIP	*x A*		*y B*		*x A B*	*B*	*A*	*A* ≈ *B*
Experiment 2								
RR	*A B*	1 s	—	1, 10 s	*A B*	—	*A B*	*A B* > *C D*
	—		*C D*		*C D*	*C D*	*C D*	
								
OIP	*B A*		*B A*			*A B*	—	*A B* > *C D*
	*C D*		*C D*			*C D*	*C D*	
								
RR + OIP	*B A*		—			—	—	*A B* >> *C D*
	—		*C D*			*C D*	*C D*	
								
RR − OIP	*A B*		—			—	*A B*	*A B* ≈ *C D*
	—		*D C*		*A*	*C D*	—	
*Note.* RR = relative recency; OIP = object-in-place; ISI = inter-sample interval; SGP = self-generated priming; RGP = retrieval-generated priming. *A*, *B*, *C*, and *D* denote target images, presented to participants on a computer display. For Experiment 1, *x*, *y* were contextual stimuli, and in all conditions *A* is presented in the first sample phase, and *B* in the second. In the *xAB* and *yAB* conditions *x* is presented with *A*, and *y* with *B*, during the sample phases. In all conditions, participants are tested with *AB*; in the *xAB* condition *x* is also present at test, while *y* is present in the *yAB* condition. In Experiment 2 in the RR condition participants exposed to *AB* followed by *CD* and then tested with *ABCD*; all images are tested in their preexposure locations. In the OIP condition participants are exposed twice to *BACD* and at test one pair of images, in the example above *AB*, changes location. The RR + OIP and RR − OIP conditions are identical to the RR condition, except that the first- or second-presented pair of images change location for RR + OIP and RR – OIP, respectively. The “SGP?” and “RGP?” columns specify the images that will be viewed less according to these respective mechanisms, and the resultant predictions of test performance is in the “Net bias?” column.								

**Table 2 tbl2:** Sample Phase: Means (and Standard Deviations) of Percent Time Gaze per AOI (Gaze Scores) Corresponding to the First- and Second-Presented Images During the A and B Sample Phases of Experiment 1; Means Are Presented Separately for Each Trial Type and Each Delay

Condition	Delay (s)	Sample	*M*	*SD*
RR	0.5	A	67.93	17.36
		B	67.11	20.34
	1	A	69.10	15.14
		B	69.33	15.79
	2	A	69.36	15.84
		B	70.52	14.18
RR + OIP	0.5	A	51.99	15.84
		B	53.85	15.97
	1	A	51.88	14.77
		B	54.42	16.50
	2	A	51.82	12.61
		B	55.04	15.69
RR − OIP	0.5	A	51.73	13.01
		B	54.75	16.01
	1	A	51.53	14.63
		B	53.24	14.97
	2	A	52.31	15.98
		B	54.17	16.66
*Note.* AIO = areas of interest; RR = relative recency; OIP = object-in-place.				

**Table 3 tbl3:** Sample Phase: Means (and Standard Deviations) of Percent Time Gaze per AOI (Gaze Scores) Corresponding to the First- and Second-Presented Images in the Sample Phases of Experiment 2; Means are Presented Separately for Each Trial Type and Each Retention Interval

Trial type	Retention interval	Sample	*M*	*SD*
OIP	1	1	19.13	2.84
		2	19.06	3.19
	10	1	18.85	3.06
		2	19.00	3.39
RR	1	1	37.99	5.91
		2	38.80	5.28
	10	1	38.25	5.14
		2	39.08	5.93
RR + OIP	1	1	38.08	5.58
		2	38.63	6.15
	10	1	37.86	5.60
		2	38.45	6.71
RR − OIP	1	1	37.15	6.87
		2	38.47	6.21
	10	1	38.01	6.13
		2	38.74	5.92
*Note.* AIO = areas of interest; RR = relative recency; OIP = object-in-place.				

## References

[c1] Aggleton J. P., & Brown M. W. (2006). Interleaving brain systems for episodic and recognition memory. Trends in Cognitive Sciences, 10, 455–463. 10.1016/j.tics.2006.08.003 16935547

[c2] Barker G. R. I., Evuarherhe O., & Warburton E. C. (2019). Remembering the order of serially presented objects: A matter of time? Brain and Neuroscience Advances. Advance online publication 10.1177/2398212819883088 PMC6820123 31815187

[c55] Berry C. J., Shanks D. R., Speekenbrink M., & Henson R. N. A. (2012). Models of recognition, repetition priming, and fluency: Exploring a new framework. Psychological Review, 119, 40–79. 22022831 10.1037/a0025464

[c3] Bouton M. E. (1993). Context, time, and memory retrieval in the interference paradigms of Pavlovian learning. Psychological Bulletin, 114, 80–99. 10.1037/0033-2909.114.1.80 8346330

[c4] Brodeur M. B., Dionne-Dostie E., Montreuil T., & Lepage M. (2010). The Bank of Standardized Stimuli (BOSS), a new set of 480 normative photos of objects to be used as visual stimuli in cognitive research. PLoS ONE, 5, e10773 10.1371/journal.pone.0010773 20532245 PMC2879426

[c5] Brodeur M. B., Guérard K., & Bouras M. (2014). Bank of Standardized Stimuli (BOSS) phase II: 930 new normative photos. PLoS ONE, 9, e106953 10.1371/journal.pone.0106953 25211489 PMC4161371

[c6] Carpenter R. H. S. (1988). Movements of the eyes (2nd ed.). London, UK: Pion.

[c7] Cowell R. A., Bussey T. J., & Saksida L. M. (2006). Why does brain damage impair memory? A connectionist model of object recognition memory in perirhinal cortex. The Journal of Neuroscience, 26, 12186–12197. 10.1523/JNEUROSCI.2818-06.2006 17122043 PMC6675420

[c8] Crutcher M. D., Calhoun-Haney R., Manzanares C. M., Lah J. J., Levey A. I., & Zola S. M. (2009). Eye tracking during a visual paired comparison task as a predictor of early dementia. American Journal of Alzheimer’s Disease and Other Dementias, 24, 258–266. 10.1177/1533317509332093 PMC2701976 19246573

[c9] Dix S. L., & Aggleton J. P. (1999). Extending the spontaneous preference test of recognition: Evidence of object-location and object-context recognition. Behavioural Brain Research, 99, 191–200. 10.1016/S0166-4328(98)00079-5 10512585

[c10] Ennaceur A., & Delacour J. (1988). A new one-trial test for neurobiological studies of memory in rats. 1: Behavioral data. Behavioural Brain Research, 31, 47–59. 10.1016/0166-4328(88)90157-X 3228475

[c11] Fischer B., Smith M., & Pau G. (2019). rhdf5: R Interface to HDF5 (R package version 2.28.1) [Computer software]. Retrieved from https://github.com/grimbough/rhdf5

[c12] Gills J. L., Glenn J. M., Madero E. N., Bott N. T., & Gray M. (2019). Validation of a digitally delivered visual paired comparison task: Reliability and convergent validity with established cognitive tests. GeroScience, 41, 441–454. 10.1007/s11357-019-00092-0 31463649 PMC6815320

[c13] Hannula D. E., Ryan J. D., Tranel D., & Cohen N. J. (2007). Rapid onset relational memory effects are evident in eye movement behavior, but not in hippocampal amnesia. Journal of Cognitive Neuroscience, 19, 1690–1705. 10.1162/jocn.2007.19.10.1690 17854282

[c14] Hintzman D. L. (1988). Judgments of frequency and recognition memory in a multiple trace memory model. Psychological Review, 95, 528–551. 10.1037/0033-295X.95.4.528

[c15] Honey R. C., & Good M. (2000). Associative modulation of the orienting response: Distinct effects revealed by hippocampal lesions. Journal of Experimental Psychology: Animal Behavior Processes, 26, 3–14. 10.1037//0097-7403.26.1.3 10650540

[c16] Kelley K. (2007). Confidence Intervals for Standardized Effect Sizes: Theory, Application, and Implementation. Journal of Statistical Software, 20, 1–24. 10.18637/jss.v020.i08

[c17] Mahoney E. J., Kapur N., Osmon D. C., & Hannula D. E. (2018). Eye tracking as a tool for the detection of simulated memory impairment. Journal of Applied Research in Memory & Cognition, 7, 441–453. 10.1016/j.jarmac.2018.05.004

[c18] McLaren I. P. L. (1994). Estimating recency and familiarity using trace strength. British Journal of Mathematical and Statistical Psychology, 47, 227–234. 10.1111/j.2044-8317.1994.tb01035.x

[c19] McLaren I. P. L., Kaye H., & Mackintosh N. J. (1989). An associative theory of the representation of stimuli: Applications to perceptual learning and latent inhibition In Morris R. G. M. (Ed.), Parallel distributed processing: Implications for psychology and neurobiology (pp. 102–130). New York, NY: Oxford University Press.

[c20] Mitchell J. B., & Laiacona J. (1998). The medial frontal cortex and temporal memory: Tests using spontaneous exploratory behaviour in the rat. Behavioural Brain Research, 97, 107–113. 10.1016/S0166-4328(98)00032-1 9867236

[c21] Peirce J., Gray J. R., Simpson S., MacAskill M., Höchenberger R., Sogo H., . . . Lindeløv J. K. (2019). PsychoPy2: Experiments in behavior made easy. Behavior Research Methods, 51, 195–203. 10.3758/s13428-018-01193-y 30734206 PMC6420413

[c22] Richmond J., & Nelson C. A. (2009). Relational memory during infancy: Evidence from eye tracking. Developmental Science, 12, 549–556. 10.1111/j.1467-7687.2009.00795.x 19635082

[c23] Richmond J., Sowerby P., Colombo M., & Hayne H. (2004). The effect of familiarization time, retention interval, and context change on adults’ performance in the visual paired-comparison task. Developmental Psychobiology, 44, 146–155. 10.1002/dev.10161 14994266

[c24] Robinson J., & Bonardi C. (2015). An associative analysis of object memory. Behavioural Brain Research, 285, 1–9. 10.1016/j.bbr.2014.10.046 25446743

[c25] Ryan J. D., & Cohen N. J. (2004). The nature of change detection and online representations of scenes. Journal of Experimental Psychology: Human Perception and Performance, 30, 988–1015. 10.1037/0096-1523.30.5.988 15462635

[c26] Sanderson D. J., & Bannerman D. M. (2011). Competitive short-term and long-term memory processes in spatial habituation. Journal of Experimental Psychology: Animal Behavior Processes, 37, 189–199. 10.1037/a0021461 21319917 PMC3085505

[c27] Squire L. R. (1994). Declarative and nondeclarative memory: Multiple brain systems supporting learning and memory In Schachter D. L. & Tulving E. (Eds.), Memory systems (pp. 203–231). Cambridge, MA: MIT Press. 10.1162/jocn.1992.4.3.232 23964880

[c28] Tam S. K. E., Bonardi C., & Robinson J. (2015). Relative recency influences object-in-context memory. Behavioural Brain Research, 281, 250–257. 10.1016/j.bbr.2014.12.024 25546721 PMC4318627

[c29] Tam S. K. E., Robinson J. J., Jennings D., & Bonardi C. (2014). Dissociations in the effect of delay on object recognition and the effect of dorsal hippocampal damage: Evidence for an associative model of recognition memory. Journal of Experimental Psychology, 40, 106–111. 10.1037/xan0000003 24000908

[c30] Wagner A. R. (1981). SOP: A model of automatic memory processing in animals In Miller N. E. & Spear R. R. (Eds.), Information processes in animals: Memory mechanisms (pp. 5–47). Hillsdale, NJ: Erlbaum.

[c31] Wagner A. R., & Brandon S. E. (1989). Evolution of a structured connectionist model of Pavlovian conditioning (AESOP) In Klein S. B. & Mowrer R. R. (Eds.), Contemporary Learning theories II: Instrumental conditioning theory (pp. 149–189). Hillsdale, NJ: Erlbaum.

[c32] Whitehead J. C., Li L., McQuiggan D. A., Gambino S. A., Binns M. A., & Ryan J. D. (2018). Portable eyetracking-based assessment of memory decline. Journal of Clinical and Experimental Neuropsychology, 40, 904–916. 10.1080/13803395.2018.1444737 29547067

[c33] Whitt E., Haselgrove M., & Robinson J. (2012). Indirect object recognition: Evidence for associative processes in recognition memory. Journal of Experimental Psychology: Animal Behavior Processes, 38, 74–83. 10.1037/a0025886 22103695

[c34] Whitt E., & Robinson J. (2013). Improved spontaneous object recognition following spaced preexposure trials: Evidence for an associative account of recognition memory. Journal of Experimental Psychology: Animal Behavior Processes, 39, 174–179. 10.1037/a0031344 23421400

[c35] Willenbockel V., Sadr J., Fiset D., Horne G. O., Gosselin F., & Tanaka J. W. (2010). The shine toolbox for controlling low-level image properties. Journal of Vision, 10(7), 653 10.1167/10.7.653 20805589

[c36] Yeung L.-K., Olsen R. K., Hong B., Mihajlovic V., D’Angelo M. C., Kacollja A., . . . Barense M. D. (2019). Object-in-place memory predicted by anterolateral entorhinal cortex and parahippocampal cortex volume in older adults. Journal of Cognitive Neuroscience, 31, 711–729. 10.1162/jocn_a_01385 30822207

[c37] Yonelinas A. P. (2002). The nature of recollection and familiarity: A review of 30 years of research. Journal of Memory and Language, 46, 441–517. 10.1006/jmla.2002.2864

[c38] Zola S. M., Manzanares C. M., Clopton P., Lah J. J., & Levey A. I. (2013). A behavioral task predicts conversion to mild cognitive impairment and Alzheimer’s disease. American Journal of Alzheimer’s Disease and Other Dementias, 28, 179–184. 10.1177/1533317512470484 PMC3670591 23271330

